# Using an episodic specificity induction to improve children’s future thinking

**DOI:** 10.3389/fpsyg.2023.1249090

**Published:** 2023-10-19

**Authors:** Annick F. N. Tanguay, Olivia Gardam, Jane Archibald, Gladys Ayson, Cristina M. Atance

**Affiliations:** School of Psychology, Faculty of Social Sciences, University of Ottawa, Ottawa, ON, Canada

**Keywords:** episodic specificity induction, episodic memory, future thinking, cognitive development, delay of gratification, prospective memory, planning

## Abstract

Episodic future thinking (EFT) is the ability to subjectively pre-experience a specific future event. Future-oriented cognition in young children positively predicts physical health and financial status later in life. Can EFT be improved in children, even temporarily? Developmental research emphasizes the importance of thinking about one’s own near future to enhance EFT, whereas research in adults suggests benefits reside in constructing a richly detailed event. We bridged the two perspectives to examine whether a procedure, the “episodic specificity induction” (ESI), could be adapted to encourage an episodic mode of thinking in children, benefitting performance on a variety of subsequent EFT tasks. The present study implemented a child-friendly ESI in which children mentally simulated a future event and were probed for specific details about it. We randomly assigned 66 children aged 6 and 7 years to one of two conditions: (1) ESI, in which children imagined “having breakfast tomorrow” in detail, describing surroundings, people, and actions, or (2) a Control condition (i.e., no construction), in which children simply viewed and described a picture of another child having breakfast. Children then completed a series of future thinking tasks assessing prospective memory, recollection/imagination of events, delay of gratification, and planning. Our ESI was successful in promoting the construction of a detailed event, and subsequently increasing the number of details of recollected and imagined events on an outcome task as compared to a control condition. Nonetheless, the effect of ESI was smaller than expected – a finding that fits with recent work suggesting that such interventions may be too cognitively taxing for young children and/or that benefits may hinge on further development in episodic processes. We discuss possible modifications to the induction and implications for EFT amelioration in young children.

## 1. Introduction

A large proportion of adults’ thoughts turn to the future on a day-to-day basis (Cole et al., 2016), such as when planning a work meeting, an upcoming first date, or a trip to the grocery store. Some of these thoughts can be broad and unspecific (e.g., ‘I will go to Disneyland every year one day’), whereas others can be very specific, enabling us to foresee the unfolding of a day in minute details (e.g., ‘On my next birthday, my four best friends and I will visit Canada’s Wonderland. We will excitedly wait in line to try out a new rollercoaster’; Szpunar et al., 2014). Knowledge about the future –as in the former case– is termed *semantic future thinking*, whereas the simulation of a specific event in one’s own future –as in the latter case– is termed *episodic future thinking* (EFT; Atance and O’Neill, 2001). Projecting into the future to simulate specific events allows us to “pre-feel” or “pre-experience” future emotional, physiological, and cognitive states (Gilbert and Wilson, 2007; Szpunar et al., 2014). Future thinking holds several benefits (Schacter et al., 2017) for our decision-making and saving behavior (Ballance et al., 2022), molding personal identity (D’Argembeau et al., 2012; Barsics et al., 2016), regulating emotions (Barsics et al., 2016; Duffy and Cole, 2021), remembering to perform an action (Altgassen et al., 2017), and possibly even promoting academic success (Prabhakar et al., 2016). The adaptive function of future thinking is such that some consider it to have shaped human evolution (Suddendorf and Corballis, 1997) and constitutes the *raison-d’être* of the ability to remember the past (Schacter et al., 2007).

Future thinking is highly adaptive and hence, unsurprisingly, researchers have tried to implement interventions to improve it. One stream of research relies on the theoretical importance of constructive episodic simulation (a flexible [re]combination of episodic details) for episodic future thinking (Schacter and Madore, 2016). Indeed, a manipulation called the “Episodic Specificity Induction” (ESI), heightens an episodic retrieval mode and brain activity in the core network (Madore et al., 2016b). This enhanced function can then ripple to subsequent tasks, including those involving problem-solving (Jing et al., 2016), creativity (Madore et al., 2015, 2016a), emotion regulation (Jing et al., 2016, 2017), and the richness of recall and future imagination (Madore et al., 2019). The ESI can entail the vivid recollection of a lab-based episodic memory (i.e., a video; Madore et al., 2014) or an autobiographical event, or the vivid imagination of a personal future event (Madore et al., 2019), all supported through a procedure inspired from the Cognitive Interview (Fisher and Geiselman, 1992). Although past- and future-oriented inductions can both produce benefits, performance on the outcome tasks may be maximized when these tasks and the induction share the same temporal orientation (e.g., a near future induction and a near future outcome task; Hallford et al., 2022).

Future thinking is also highly adaptative for children. Might prospection also be ameliorated, even on a temporary basis, in this population? Most of the work on children’s EFT has attempted to measure the ability, identify developmental milestones, and understand its relation to other cognitive abilities (reviewed in Atance, 2015). Although children’s thoughts may be less frequently future-oriented than adults’ (McCormack et al., 2019), children can still spontaneously talk about the future when trying to allocate resources in an optimal manner, and perform better when they do (Caza and Atance, 2019). As such, might a brief activity to *prompt* children’s talk about the future in turn benefit a host of future-oriented behaviors, including episodic simulation, prospective memory, anticipation of future needs, and delay of gratification? On the one hand, developmental research suggests that thinking about one’s own future self – even in general terms – can improve EFT in children (e.g., ‘I will have breakfast tomorrow morning’; Chernyak et al., 2017; Leech et al., 2019). On the other hand, ESI research in adults indicates that engaging constructive processes through the (re)combination of a multiplicity of episodic elements can enhance many related cognitive functions, including EFT itself (Madore et al., 2016a, 2019). Therefore, in the current study, we examine whether encouraging a detailed or episodic-like imagination of the future, *via* a child-adaptation of the ESI (e.g., ‘For breakfast tomorrow, I will eat a waffle that is going to have a smiley face with raspberries for its eyes. Its smile is going to be made of whipped cream. And it’s going to make me laugh.’) benefits EFT in children.

Childhood offers a unique backdrop to situate the challenges (e.g., the ongoing development of many cognitive abilities along with future thinking) and opportunities (e.g., possible lifelong benefits) of future thinking interventions. With age, children sharpen their ability to describe plausible future events (Quon and Atance, 2010; Coughlin et al., 2014), envision their future preferences (Bélanger et al., 2014), anticipate future needs (Atance and Meltzoff, 2005; Suddendorf et al., 2011; Ferretti et al., 2018), delay gratification (Mischel and Metzner, 1962; Mahy et al., 2020), and remember to perform an action (Mahy et al., 2014). The development of future thinking during preschool years continues throughout childhood and adolescence (Willoughby et al., 2012; Gott and Lah, 2014; Loose and Vásquez-Echeverría, 2022).

One of the most common assessments of EFT in adults, also used in children, entails prompting a mental simulation and verbal description of a specific event that could plausibly happen in the future (Levine et al., 2002; Addis et al., 2008; Willoughby et al., 2012). The verbal responses are then scored for their episodic quality or parsed for the number and type of details (Coughlin et al., 2014, 2019; Wang et al., 2014). School-aged children recount less detailed or episodic-like events than adults (Coughlin et al., 2014, 2019; Wang et al., 2014). Similarly, older children sometimes narrate more episodic-like events than younger children (Coughlin et al., 2014), as adolescents do relative to their younger peers (Willoughby et al., 2012). Recollection of past events follows a somewhat parallel trajectory (Busby and Suddendorf, 2005; Hayne et al., 2011; Coughlin et al., 2014), consistent with a strong association between episodic memory and EFT (Addis, 2018).

Several tasks assess EFT development in childhood while attempting to avoid its conflation with language by minimally relying on verbal responses (reviewed in Atance, 2018). Such tasks include the Picture-book task, Prospective memory, and Delay of gratification. The Picture-book task is meant to assess children’s ability to infer future states by showing them different scenes (e.g., road, stream, snow) and asking them to pretend they will visit them in the future (Atance and Meltzoff, 2005). The scenarios are meant to evoke a corresponding physiological need, such as the possibility of getting thirsty, getting hurt, or getting cold (Atance and Meltzoff, 2005). Successful task completion requires anticipating the physiological state in question (e.g., getting cold) to then select the most appropriate object from a set (e.g., winter coat, bathing suit, ice cubes). Children’s performance improves between 3 and 5 years old (Atance and Meltzoff, 2005), and from 6 and 7 to 8 years old (Ferretti et al., 2018).

Although not categorized as EFT tasks *per se*, Prospective memory and Delay of gratification engage future-oriented reasoning in ways argued to benefit function in daily life (e.g., academic success, health-related behavior; Mahy et al., 2014; Daniel et al., 2015). Prospective memory, or remembering to perform an action in the future (e.g., a child reminding the researcher to give them a gift at the end of the study), matures from childhood to adulthood (Mahy et al., 2014). Similarly, older children display a better ability to delay gratification than younger children (Mischel and Metzner, 1962; Hudson et al., 2011; Mahy et al., 2020). Delay of gratification can be assessed by offering children the choice between a smaller immediate reward (e.g., one marshmallow, sticker, or pretzel) or a larger reward later (e.g., 6 stickers; Prencipe and Zelazo, 2005). In sum, children hone their future-oriented reasoning throughout childhood, as reflected in multiple cognitive domains and a variety of tasks. Critically, from a methodological standpoint, these various tasks could be sensitive to changes initiated by an activity designed to improve EFT.

Previous research suggests EFT is malleable and amenable to amelioration in young children (e.g., Chernyak et al., 2017; Leech et al., 2019; Wang et al., 2019). For example, one training instructed parents to ask their child questions about their thoughts, feelings, and desires during past events when recollecting these events over a 6-month period (Wang et al., 2019). The added focus on the children’s cognitive and affective experiences during past events sufficed to encourage a more specific imagination of future events following the intervention (Wang et al., 2019). Briefer interventions have also been shown to have beneficial downstream effects on EFT (Chernyak et al., 2017; Leech et al., 2019). These shorter activities centered on drawing a picture or reading a book and some related questions. Chernyak et al. (2017) asked children to draw themselves at a specific point in time (present, near past, near future, or distant future) and then asked them a few questions about activities (near past, near future, or distant future) or sensations/perceptions (present). Leech et al. (2019), instead, changed the tense (present or future) and depiction of characters (self or other) in a story. These various task versions helped to disambiguate the key factors underlying EFT improvements among a few candidates: self-reference (e.g., thinking about the self or another child), temporal orientation (e.g., thinking about the past, present, or future), temporal distance (e.g., projecting to a near or distant time), or a combination of these. Children performed best on the Prospective memory and/or Picture-book tasks when thinking about their own self in the near future (Chernyak et al., 2017; Leech et al., 2019) and in the near past (Chernyak et al., 2017). Thus, the conjunction of thinking about the self and a proximal temporal distance appears optimal to foster benefits on EFT in children.

Leech et al. (2019) proposed that the vividness of the extended self might determine boosts on EFT tasks. We propose another somewhat broader hypothesis drawing from the adult literature on the ESI (Schacter and Madore, 2016; Madore et al., 2019) and episodic memory: the vividness of the events *overall*, not just the representation of the self, might determine the extent of EFT benefits on subsequent tasks. Indeed, events tend to be recalled with greater details in the near past/future (D’Argembeau and Van der Linden, 2004; Berntsen and Bohn, 2010) and when personal rather than about someone else (de Vito et al., 2012; Grysman et al., 2013; Verfaellie et al., 2019), consistent with studies on brain activity (Addis et al., 2004; Tanguay et al., 2020). Therefore, a conversation or story about the self within the near past or future might enhance children’s ability to engage constructive processes. The potential role of episodic processes in Chernyak et al. (2017) and Leech et al. (2019) remains ambiguous, however, because children were encouraged to consider a few elements or facts about routine/script-like events but were not prompted to generate a highly specific and detailed event. Even the gist recall of repeated events can evoke visual details and a scene, but the amount of details may not always be maximal relative to an event that is specific in time and place (Addis et al., 2011; Tanguay et al., 2022). Hence, we adapted the ESI to test whether eliciting episodic details from children about a *specific* event would improve their EFT.

Our study investigates the potential benefits on EFT of an ESI adapted for school-aged children and online administration (due to the COVID-19 pandemic). We recruited a somewhat older sample than Chernyak et al. (2017) and Leech et al. (2019) (6–7 years old vs. 3–5 years old) to ensure children could fully partake in this online ESI. The predominant focus of our ESI was the elaboration of a highly vivid future event (i.e., tomorrow’s breakfast), because previous studies have already demonstrated that thinking about the future, even in general terms, can produce gains on EFT tasks (Chernyak et al., 2017; Leech et al., 2019). Importantly, research on the Cognitive Interview in children (Memon et al., 2010; Verkampt et al., 2014) provides provisional reassurance that an ESI procedure can be adapted for young children. We investigated whether our adapted ESI could enhance a range of future-oriented abilities (i.e., recollection/imagination of an event, delay of gratification, anticipating future states, and prospective memory). We compared ESI to a control condition that entailed describing a picture (i.e., a child having breakfast). Thus, the control condition involved no episodic (re)construction of an event, but had a similar format and content. The developmental and adult literature (reviewed above) converge to suggest that children in the ESI condition will perform better than children in the control condition on EFT tasks. Based on Chernyak et al. (2017) and Leech et al.’s (2019) results, we expected greater differences between the two groups on the Picture-book and Prospective memory tasks compared to the Delay of gratification task. In addition, based on Madore et al.’s (2019) findings, we expected to see the greatest differences between the two groups on the amount of details produced in verbal description of past and future events. More precisely, we hypothesized that the greatest differences between the two conditions would be found for the imagination of an event in the near future, which temporally matched the ESI (Hallford et al., 2022). Our study’s main hypotheses and analyses were pre-registered on the Open Science Framework.^1^

## 2. Methods

### 2.1. Participants

Seventy typically-developing 6- and 7-year-olds participated in the study (*M* age = 83.00 months, *SD* = 6.62, range = 72.41–95.51 months, 33 boys and 37 girls; 3 parents did not report the date of birth). We excluded data from four participants because: (1) two children wished to end the induction before its completion (one in each condition), (2) one was ineligible due to age, and (3) one had already participated during the piloting stage. After exclusion, the sample was composed of 34 children randomized to the control condition and 32 children randomized to the ESI condition. An independent sample *t*-test showed that the two groups did not differ on age in months, *t*(62) = 0.22, *p* = 0.829, Hedges’ *g* = 0.05, CI 95% [−0.48, 0.57], and a Chi-square test with continuity correction found no significant difference in gender, *Χ*^2^ (1, *N* = 66) = 3.12, *p* = 0.077, φ = 0.22. All children were fluent in English (see Table 1).

**Table 1:** Demographic characteristics per condition.

	Age (in months)^1^	Gender	Ethnicity (% of subgroup)^2^	Yearly household income (% of subgroup)^3^	English as first language^4^
Control (*n* = 34)	*M* = 82.98, *SD* = 5.97, range = 72.41–93.47	19 boys, 15 girls	50% White, 5.88% Black and White, 2.94% (*n* = 1) each: Canadian, Indian (Asia), Black, British White, Canadian and White, European, Filipino, White and Asian, White and Indian (Asia), Indian (Asia) and Lebanese, White and Lebanese, 11.76% (*n* = 4) not reported	> $100,000: 73.53% ≤ $100,000: 17.65%Not reported: 8.82% (*n* = 3)	82.35 (8.82% other language, 8.82% [*n* = 3] not reported)
ESI (*n* = 32)	*M* = 82.62, *SD* = 7.31, range = 72.67–95.38	10 boys, 22 girls	62.50% White, 3.13% (*n* = 1) each: Mixed, Canadian, South Asian, Latin White, Chinese, Greek Lebanese, Jewish, Serbian Canadian, 12.50% (*n* = 4) not reported	> $100,000: 62.50% ≤ $100,000: 21.88%Not reported: 15.63% (*n* = 5)	84.38% (3.13% other language, 12.50% [*n* = 4] not reported)

^1^64 children had a precise date of birth. ^2^58 parents reported the ethnicity of their child. The table summarizes ethnicity based on parents’ preferred label. ^3^58 parents reported income. ^4^59 parents reported information on language.

Our sample size was determined through an *a priori* G*Power 3.1 (Faul et al., 2009) analysis using Chernyak et al. (2017) to estimate the effect size (*t*-test, difference between two independent means, two-tailed, *d* = 0.5, *p* = 0.05, ß = 0.80) which suggested a sample size of *N* = 66. Children were recruited through email from participant databases associated with University of Ottawa’s Child Development Laboratories and tested online on Zoom (zoom.us). Children received a $10 gift card for their participation. The ethical aspects of the study were reviewed by the Health Sciences and Sciences Research Ethics Board of the University of Ottawa (file # H-02-19-2769). Many constraints arose from aiming to offer a safe and positive research opportunity to children who completed the study from their home during the COVID-19 pandemic (from December 2020 to May 2021). Even though online administration introduced challenges (e.g., computers and the home environment can be sources of distraction), it was closer to the optimal format of an ESI activity in children. That is, we endeavored to develop an ESI that could be administered with minimal training, at home or in school settings.

### 2.2. Procedure

Two female researchers collected data remotely over the Zoom video-conferencing platform. We obtained verbal consent from the parents and verbal assent from children. We asked parents to remain available throughout the session to assist with any technological issues, while emphasizing the importance of not interacting with their child. We also specified that the session should take place away from the kitchen or dining room to minimize risks of “breakfast” cues being immediately available in the environment. This was possible for most but not all children, due to constraints such as space configuration or family members using other rooms. After the ESI or control condition (described next), the outcome tasks unfolded in this order: Instructions for the Prospective memory task, the Recollection/Imagination task, Delay of gratification task, Picture-book task and, if necessary, a Prospective memory reminder. The order was fixed to introduce enough time between instructions for the Prospective memory task and the end of the session, and between the Delay of gratification task and receipt of the “delayed” reward if chosen by the child. Further, the Recollection/Imagination task was always administered second, because a similar outcome task directly followed the adult ESI in Madore et al. (2019) and showed the benefits of ESI. We administered the Delay of gratification task and Picture-book task via Gorilla.sc (Anwyl-Irvine et al., 2020) to minimize experimenter influence – who were not blind to the participants’ experimental conditions. A scripted interaction with the researcher was necessary for the Recollection/Imagination task and Prospective memory task (as described below).

#### 2.2.1. Experimental manipulation

We randomly assigned children to an ESI or control condition. Both conditions were designed to last approximately 10 min.

##### 2.2.1.1. Episodic specificity induction condition

Our ESI was based on Madore et al.’s (2019) *Imagination specificity induction*. Our version began with the researcher telling children that they were going to play an imagination game and that they were going to imagine that they would be “*having breakfast tomorrow.*” We specified the event, rather than allowing children to freely select one, because Atance (2018) argued that children describe their futures more accurately when they are given a specific event to discuss. Moreover, parents consider their children to have a high level of control over their breakfast experiences (Quon and Atance, 2010). Further, the lockdowns and other COVID-related safety measures limited the episodic details and type of events that were probable. Breakfast was, of course, a probable event throughout the pandemic. Based on the child Cognitive Interview (and adult Cognitive Interview/ESI; Memon et al., 2010; Madore et al., 2019), participants were told they could imagine anything as they “*know best what could happen*,” and that they could say “*whatever comes to your head*.” These instructions were important to ensure that children felt comfortable sharing anything that they were imagining. Children were then informed that closing their eyes helps to imagine and the researcher demonstrated briefly how to imagine something with your eyes closed (e.g., “*I’m imagining myself watching TV at home, my hair is in a ponytail, and I’m wearing a green shirt and blue pants*”). The researcher then showed the children a picture of herself with a thought bubble representing the scene.

Next, children were instructed to close their eyes while the researcher asked them questions, and to picture what they were imagining in their head. The researcher then asked the children to imagine themselves having breakfast tomorrow and asked them a series of open-ended questions about (1) surroundings (e.g., “*Tell me where you will be.*”), (2) people (e.g., “*What will you be wearing?*,” “*What will people around you be wearing*?”), and (3) actions (e.g., “*Tell me everything that will be happening.*”; see Supplementary Table 1). Unlike in Madore et al.’s (2019) ESI, we did not encourage children to freely describe the event at the beginning of the induction but, rather, prompted for different kinds of details. This decision was made to avoid taxing cognitive processes other than those required for episodic projection into the future.

After each of the initial questions, the researcher asked children follow-up questions to prompt for as many details as possible to ensure the child was creating a vivid image of the event (e.g., “*You mentioned an (object), what color is it?*”; “*Is there anything on the walls?*”). If children did not respond to a question, they were reminded to close their eyes (if they had opened them), and that they could imagine anything. The initial question was then repeated. A decision tree was used during questioning so that the researcher knew when to move on to the next question, and to ensure that the ESI was administered in a standardized way (see Supplementary Table 1 for a simplified version of the decision tree). The decision tree included guidelines on what should be considered a sufficiently detailed response.

##### 2.2.1.2. Control condition

In contrast to the ESI, this condition was designed to be “non-episodic” and non-constructive in nature. Children were told they were going to play a picture game and that they would have to answer questions about a picture. First, the researcher described a simple picture, shown on the screen, to demonstrate to the child what they would have to do (e.g., “*In this picture, I see a girl holding a book, she is wearing a blue shirt, and pink skirt,*” or equivalent for boys). They were then presented with a picture of a child having breakfast and were asked questions about the picture. As in the ESI, questions were about surroundings (e.g., “*Where is the boy/girl?*”), people (e.g., “*What is the boy/girl wearing?*”), and actions (e.g., “*What is happening in the picture?*”; see Supplementary Table 1 for a full list of questions). Further prompts were given if necessary, so that the child provided as much detail about the picture as possible. If children’s responses began to stray too far from what could be seen in the picture, children were encouraged to only describe what they saw, and the researcher moved on to the next question. We aimed to match the duration of the overall manipulation rather than the number of questions per section (surroundings, people, actions) because the theme of “actions” was less relevant to an image. The goal of this condition was providing an activity with a similar format and content to the ESI (i.e., a structured interview about breakfast). In order to achieve the level of details criteria, ESI and Control inductions were not time limited, but the researchers aimed to complete the inductions within approximately 10 min.

##### 2.2.1.3. Visual clarity scale

After each of the three ESI/control condition sections (i.e., surroundings, people, actions), the researcher showed children the Visual Clarity Scale (see Figure 1 in Coughlin et al., 2014) and asked “*When you thought about your (surroundings, appearance, or actions), how clear did it look in your head?*” (for the ESI condition), and “*When you looked at the picture, how clearly could you see (what was in the room, how the boy/girl looked, what was happening in the picture)?*” (for the control condition). Children could choose from one of six options, each corresponding to an image (not clear at all, pretty unclear, not so clear, somewhat clear, very clear, or perfectly clear; as in Coughlin et al., 2014).

#### 2.2.2. Outcome measures

##### 2.2.2.1. Prospective memory task

The instructions for this task were administered immediately after either the ESI or the Control condition [inspired from Chernyak et al. (2017) and Guajardo and Best (2000)]. The researcher asked children to think of a favorite thing (e.g., stuffed animal, toy, book) that they would like to show the researcher at the end of the games. Once the child thought of an object, the researcher told the child, “*I would love to see this, but I have a very bad memory. I will likely forget. Can you remember to get this (object) after we finish all our games?*” The researcher then reiterated the instructions [“*So when I say, ‘We are all finished playing our games’, that’s when you should go get your (object).*”] and asked the child to repeat the instructions back to them to ensure they understood. If the child could not remember the task, the researcher reminded them that they must get their favorite thing at the end of the games.

At the end of the session, the researcher said, “*We are all finished playing our games*” and waited 10 s to see if the child remembered to go get their object. If they did not, the researcher asked, “*Is there something you wanted to remind me of?*” After another 10 s, the researcher reminded the child to go grab their favorite thing if it had been forgotten. This task was scored out of 2, with 2 points awarded if children remembered immediately, 1 point if they remembered after the prompt, and 0 points if they did not remember at all. Data from one child was excluded because they retrieved the object before the end of the session.

##### 2.2.2.2. Recollection/imagination task

This verbal task was adapted from Coughlin et al.’s (2014) future-thinking interview and included three randomized trials with different temporal distances and orientations: near past, near future, and distant future. Children were told that they would be asked about things that happened in the past and could happen in the future, and that these things should be “specific.” The researcher provided them with an example of an appropriately specific event (“*I bought bananas at the grocery store last night*”) and an event that, in contrast, would be considered too general (“*I always buy bananas because I love them*”; inspired from Wang et al., 2014). Instructions were as follows: “*I want you to think of something that happened/could happen any time in the last week/next week/next year. Remember, it should be specific. I’m going to give you a word to help you think of something. It can be anything that the word school/family/dessert makes you think about*.” The cue words were taken from Coughlin et al.’s (2014) task (with the exception of “cake,” which we replaced with “dessert” to make the cue word more open-ended) and were randomly assigned to a trial. If the child could not think of an event, or did not understand the instructions, the researcher reformulated the question or chose from a bank of extra cue words if necessary [e.g., song, pet, book, game, also from Coughlin et al. (2014)]. Once the child thought of an event, the researcher said “*Tell me everything you can about this. Start at the beginning. What happened?*” and then asked, “*Anything else?*” if children remained silent after 5–10 s. Additionally, if the event the child provided was too general or strayed from the temporal distance or orientation, the researched asked, “*Can you tell me about a specific time when this happened/will happen last week/next week/next year?*” These trials were not time limited. The researcher moved on to the next trial once the child had nothing left to say. In some cases, children identified a specific event before the researcher had given the cue. The researcher did not interrupt children in these cases and moved on to the next trial based on the same rules. Similarly, in cases where events were unrelated to the cue, the researcher did not correct the child. We included and scored all trials, whether cue-related or not. We excluded three participants who skipped a trial and one participant whose parent suggested their response.

The Recollection/Imagination responses were transcribed following guidelines in Wardell et al. (2021) and scored by a coder who was blind to participant condition. We adapted Coughlin et al.’s (2014) scoring procedure (based on Piolino et al., 2007) to obtain an episodicity score; this procedure has been shown to be feasible for 6- and 7-year-olds. Each of the three events was given a score from 0 to 5 based on its specificity, temporal and spatial details, use of additional details, and event progression (see Supplementary Table 2, for additional details and examples). Two scorers rated 33 participants (Control: *n* = 20; ESI: *n* = 13) and obtained moderate to good/excellent agreement on each of the trials using a two-way random intraclass correlation for a single measurement, testing for consistency, for near past, 0.79, 95% [0.62, 0.89], near future 0.77, 95% [0.58, 0.88], and distant future 0.81, 95% [0.65, 0.91]. One of the 33 participants only completed the near future trial.

Additionally, responses to the Recollection/Imagination task were scored using the adapted Autobiographical Interview procedure (Levine et al., 2002; Addis et al., 2008; Willoughby et al., 2012). The AI scoring procedure has been used in adults to demonstrate the effects of ESI on episodic memory and episodic future thinking (e.g., Madore et al., 2014, 2019). For the Recollection/Imagination task, we counted details about the main event as internal details, for example the details could be about the events’ unfolding, the surroundings, time, people, and emotions and thoughts. Other details were categorized separately as “external details”; these included semantic details (e.g., “I have two sisters”), metacognitive statements (e.g., “I do not know what else”), repetitions, and details about another event than the main event. Three scorers achieved moderate to good/excellent agreement for internal details on 20 transcripts (Control: *n* = 11; ESI: *n* = 9) for each trial: near past (0.73, 95% CI = 0.53, 0.87), near future (0.89, 95% CI = 0.79, 0.95), and distant future (0.70, 95% CI = 0.50, 0.86) for the Recollection/Imagination task.

We used a similar AI scoring procedure to examine the amount of details elicited during the manipulations (i.e., ESI, Control). The scoring of ESI was similar to regular AI procedure as described above: Details about the event of having breakfast tomorrow were categorized as internal details (e.g., “I will feel happy,” “my dad will make pancakes,” “It will be early morning,” “I will eat in the living room”). Other details were categorized separately as “external details”; these included semantic details (e.g., “I always eat toasts”), metacognitive statements, repetitions, and details about another event than tomorrow’s breakfast. The main adaptation concerned the Control condition: As in Madore et al. (2019) for an analogous task, internal details referred to details about the image (e.g., “The star is yellow”), whereas external details referred to any unrelated details or inferences that could not be made solely on the basis of information in the image (e.g., “The boy is happy because it’s his birthday”). We did not consider details to be “off-task” or meta-cognitive if elicited due to technical issues, environmental distractions (e.g., an interruption from a sibling), or repetitions that were required due to the interactional and online format. We estimated that children should produce at least 30% of internal details (i.e., details involving episodic elaboration) in the ESI condition to support its potential efficacy for improving EFT. The age of children and description of a routine event motivated this relatively low threshold. We tested the consistency in the number of details between three scorers on a random subset of 10 ESI and 10 control conditions using a two-way random intraclass correlation for a single measurement and it was excellent for internal details (0.95, 95% CI = 0.90, 0.98), and moderate to excellent for external details (0.79, 95% CI = 0.62, 0.90). We conducted analyses of the episodicity score and internal details using the score of one of the scorers included in the agreement analyses. The scorer was unaware of participants’ condition and was the most experienced of the three scorers.

##### 2.2.2.3. Delay of gratification

This task, adapted for online administration using Gorilla.sc (Anwyl-Irvine et al., 2020) was meant to assess children’s ability to delay a smaller, immediate reward in favor of a larger, later reward (inspired from Prencipe and Zelazo, 2005). An audio recording informed children that they were going to play a choices game where they could choose between watching one “cute” animal video now, or several “cute” animal videos later. Children were shown what happened when the “now” option was selected (i.e., they saw one brief clip), and they were told that they would be shown more videos later, at the end of the games, if they chose “later.” The children were informed that there were no right or wrong answers and were asked to indicate whether they preferred dogs or cats so that they would be shown videos of the corresponding animal. This task contained three trials (in random order) during which children could choose to watch one video now or 2/4/6 videos later. For this and the subsequent task, children responded verbally and the researcher pressed the selected option on their own computer. Children watched the videos either immediately or at the end of the session depending on their choices. This task was scored out of 3, with 1 point given every time children chose the delayed option.

##### 2.2.2.4. Picture-book task

This task was intended to examine children’s ability to anticipate a future physiological state (Atance and Meltzoff, 2005). The task was adapted for online administration on Gorilla.sc (Anwyl-Irvine et al., 2020) and to increase difficulty given our older sample. Namely, we included 4 trials (instead of 6) and showed 4 response options for each (instead of 3; see Supplementary Table 3). At the beginning of each trial, children saw a photograph of a nature scene (a rocky stream, a waterfall, a mountain, a snow-covered path), and were told to pretend they would be visiting that location “*tomorrow*.” Each photograph was meant to evoke thoughts about a specific physiological state (e.g., a snow-covered path evokes the state of “being cold”). With the photograph of the scene on the screen, the audio recording stated, “*You can only bring one of these things there.*” And asked “*Which one do you need to bring to this place?*” The image for each response appeared one by one with concurrent audio recorded object labels. The correct item (e.g., tuque) was the one that best addressed the physiological state in question (e.g., being cold). The remaining three choices included an option that was semantically associated with the best option (e.g., baseball cap) – but that was suboptimal – and two options that were semantically associated with the scene, but inappropriate and incorrect (e.g., ice cube and snow globe). Trial order was randomized and item presentation order was pseudo-randomized with the Latin Square design. After selecting an item, the selected response option appeared on the screen and an audio recording asked children to explain their choice.

For the item choice measure, children were given a score of 0 if they chose an incorrect item and a score of 1 for the correct item on each trial, for a total score between 0 and 4. For the explanation measure, answers were transcribed and coded to determine whether children referred to the relevant future physiological state to justify their choice. Using an adaptation of Atance and Meltzoff’s (2005) scoring system, children’s responses were categorized as follows: (1) future state, meaning children referred to their future physiological state and used a future term (e.g., “I’m gonna get hungry on that walk” [3 points]); (2) future talk, meaning children used a future term without mentioning the future state (e.g., “I might need it” [2 points]); (3) non-future state, meaning children did not use a future term, but mentioned a physiological state (e.g., “Because it’s cold” [1 point]) (4) or non-future talk, meaning children referred to the item’s function without using any future-oriented terms, or gave a nonsensical explanation (e.g., “Because it’s for eating” [0 points]).

Two coders independently scored the responses on each trial for all of the children. One child opted to skip this task and was not included in any of the Picture-book task analyses. We obtained an ICC value of 0.91, 95% CI [0.86, 0.95] for the mean score obtained by children on the verbal measure based on single rating, consistency, two-way model, indicating excellent inter-rater reliability. The scores of one of these two coders were included in the analyses. The selected scorer was unaware of the participants’ condition.

### 2.3. Statistical analyses

Statistical analyses were conducted using R (R Core Team, 2022) in Rstudio (v. Rstudio 2022.07.2 + 576, and validated in 2023.06.0 + 421). For all parametric and “non-robust” tests, we minimized the influence of extreme values through winsorizing (bringing extreme values closer to the mean to take the value equivalent to *z* ± 2.58; implemented in ‘scipub’; Pagliaccio, 2022). Data were log10 transformed to obtain a normal distribution when possible. If normality could not be achieved, we presented results from a non-parametric (for *t*-tests) or a robust alternative (for mixed analysis of variance; ANOVA). Specifically, we used the ‘stats’ v. 4.2.2 package to conduct *t*-tests and Mann–Whitney tests, ‘rstatix’ package (Kassambara, 2022) for pairwise t-tests and Hedges’ *g*, ‘ez’ (Lawrence, 2016) for mixed ANOVAs, and ‘WRS2’package (Mair et al., 2022) for robust ANOVAs (with a trim level of 0.20). All tests were two-tailed and were considered statistically significant when *p* < 0.05. We applied a Holm-Bonferonni correction on post-hoc tests. We took a pairwise deletion approach, always analyzing the maximum amount of data on each task.

## 3. Results

### 3.1. Characteristics of the induction

#### 3.1.1. Duration

The ESI manipulation (log10 transformed as in analysis: *M =* 1.15, *SE* = 0.01; untransformed: *M* = 14.28 min, *SE* = 0.49, 9.88 to 21.32 min) lasted longer than the Control manipulation (transformed: *M* = 0.93, *SE* = 0.01; untransformed: *M* = 9.02 min, *SE* = 0.49, range 6.32 to 22.28 min) from the beginning of the instructions to the end of the manipulation, *t*(64) = −10.44, *p* < 0.001, Hedges’ *g* = −2.54, CI 95% [−3.37, −1.93].

#### 3.1.2. Number of details and words

Participants generated more internal (“on task”) details during the ESI manipulation (*Mdn* = 76, *M* = 76.72, *SE* = 4.80, *N* = 32) than the Control manipulation (*Mdn* = 58, *M* = 59.50 *SE* = 3.19, *N* = 34), *W* = 294, *p* = 0.001, *r* = 0.40. The greater amount of information in the ESI (*Mdn* = 417.5, *M* = 461.12, *SE* = 44.81, *N* = 32) than Control manipulation (*Mdn* = 247, *M* = 260.59, *SE* = 19.45, *N* = 34) is also supported by the participants’ number of words (estimated through word tokenization in R; De Queiroz et al., 2022), *W* = 245, *p* < 0.001, *r* = 0.47. However, children overwhelmingly replied with on-task details in both conditions (ESI: *M* = 87.16%, *SE* = 2.13, range: 51.38 to 100%; Control: *M* = 94.28%, *SE* = 1.03, range: 73.17 to 100%).

#### 3.1.3. Visual clarity ratings

As expected given the nature of both tasks (seeing a physical image vs. imagining an event in the “mind’s eye”), a Mann–Whitney test showed a significantly higher mean visual clarity rating in the Control condition (*Mdn* = 5.67, *M* = 5.41, *SE* = 0.11, *N* = 34) than in the ESI condition (*Mdn* = 5.00, *M* = 4.79, *SE* = 0.19, *N* = 32), *W* = 730.5, *p* = 0.015, *r* = 0.30. A mean rating of approximately 5 (out of 6) suggests children could visualize events that were “very clear.” The high number of internal details (reported above) and the high vividness rating combined strongly suggest that the ESI was successful. Indeed, one child even spontaneously justified the ratings saying the image was “a 3D one, where it’s really me at the table eating my pancakes.”

**Figure 1:**
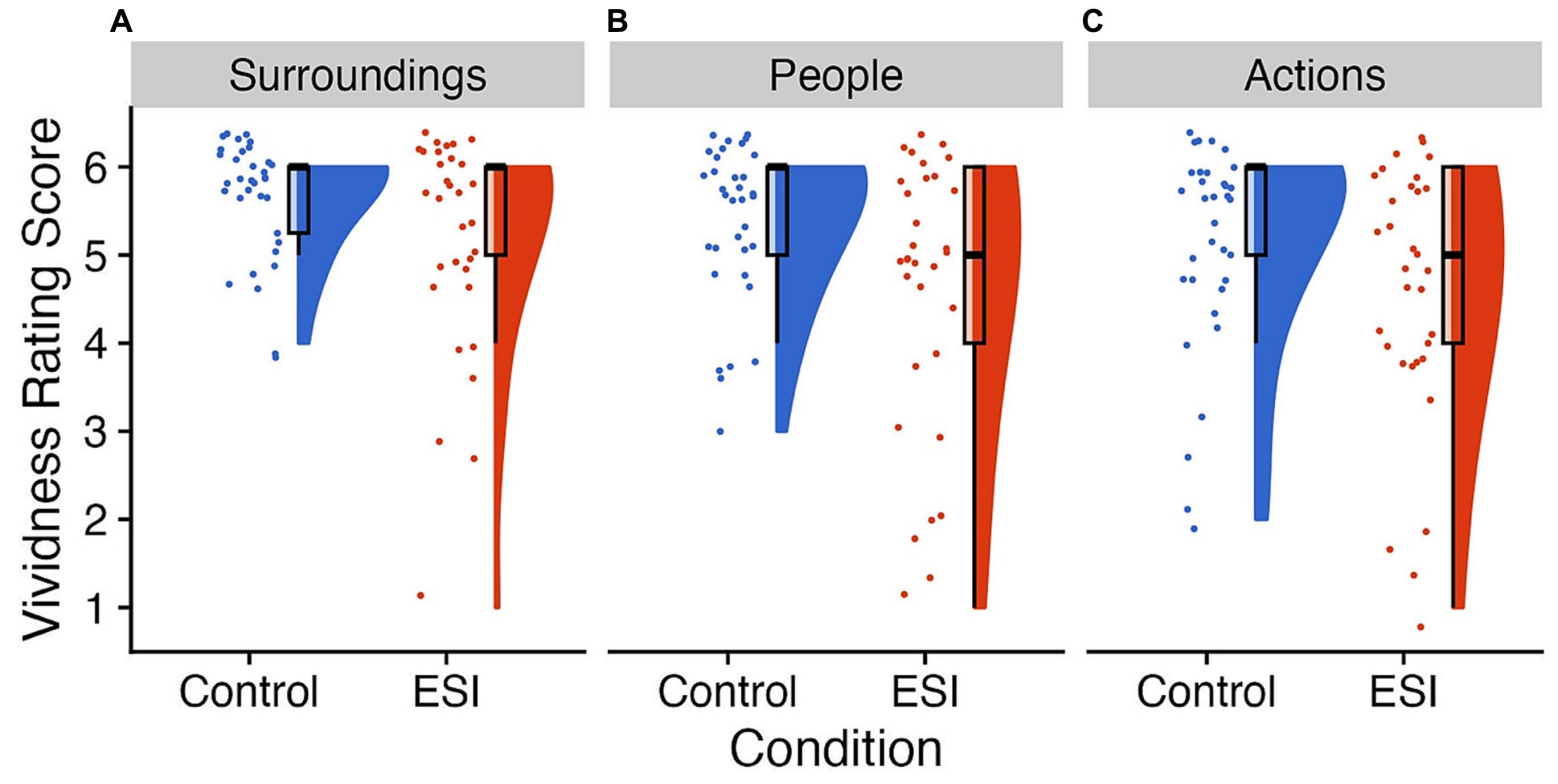
Ratings on the Visual Clarity Scale. Raincloud plots of the rating on the visual clarity scale after each section of the manipulation, **(A)** surroundings, **(B)** people, and **(C)** actions with the Control condition on the left of each panel in blue and the ESI condition on the right of each panel in orange. The “clouds” show the probability density function, overlayed with boxplots in black, whereas the “rain” (i.e., dots) represent individual data points. The individual data points were jittered for improved visualization. On the y axis, 1 = not clear at all, 2 = pretty unclear, 3 = not so clear, 4 = somewhat clear, 5 = very clear, and 6 = perfectly clear. Prepared in R using RainCloudPlots (Allen et al., 2021).

In follow-up analyses, we explored whether some sections of the ESI were particularly successful at eliciting vivid mental images in children, thus reducing (or even eliminating) the gap in visual clarity ratings between the conditions. Children in the ESI (surroundings: *Mdn* = 6, *M* = 5.19, *SE* = 0.21, *N* = 32; actions: *Mdn* = 5, *M* = 4.59, *SE* = 0.26, *N* = 32) and Control (surroundings: *Mdn* = 6, *M* = 5.68, *SE* = 0.10, *N* = 34; actions: *Mdn* = 6, *M* = 5.15, *SE* = 0.20, *N* = 34) conditions did not significantly differ in their ratings of their surroundings, *W* = 670.00, *p* = 0.059, *r* = 0.23, or actions, *W* = 672.00 *p* = 0.083, *r* = 0.21 (see Figures 1A,C). However, ratings for the “people” section of the ESI (*Mdn* = 5.00, *M* = 4.59, *SE* = 0.28, *N* = 32) was significantly lower than the Control condition (*Mdn* = 6.00, *M* = 5.41, *SE* = 0.14, *N* = 34), *W* = 709.50, *p* = 0.023, *r* = 0.28 (see Figure 1B).

**Figure 2:**
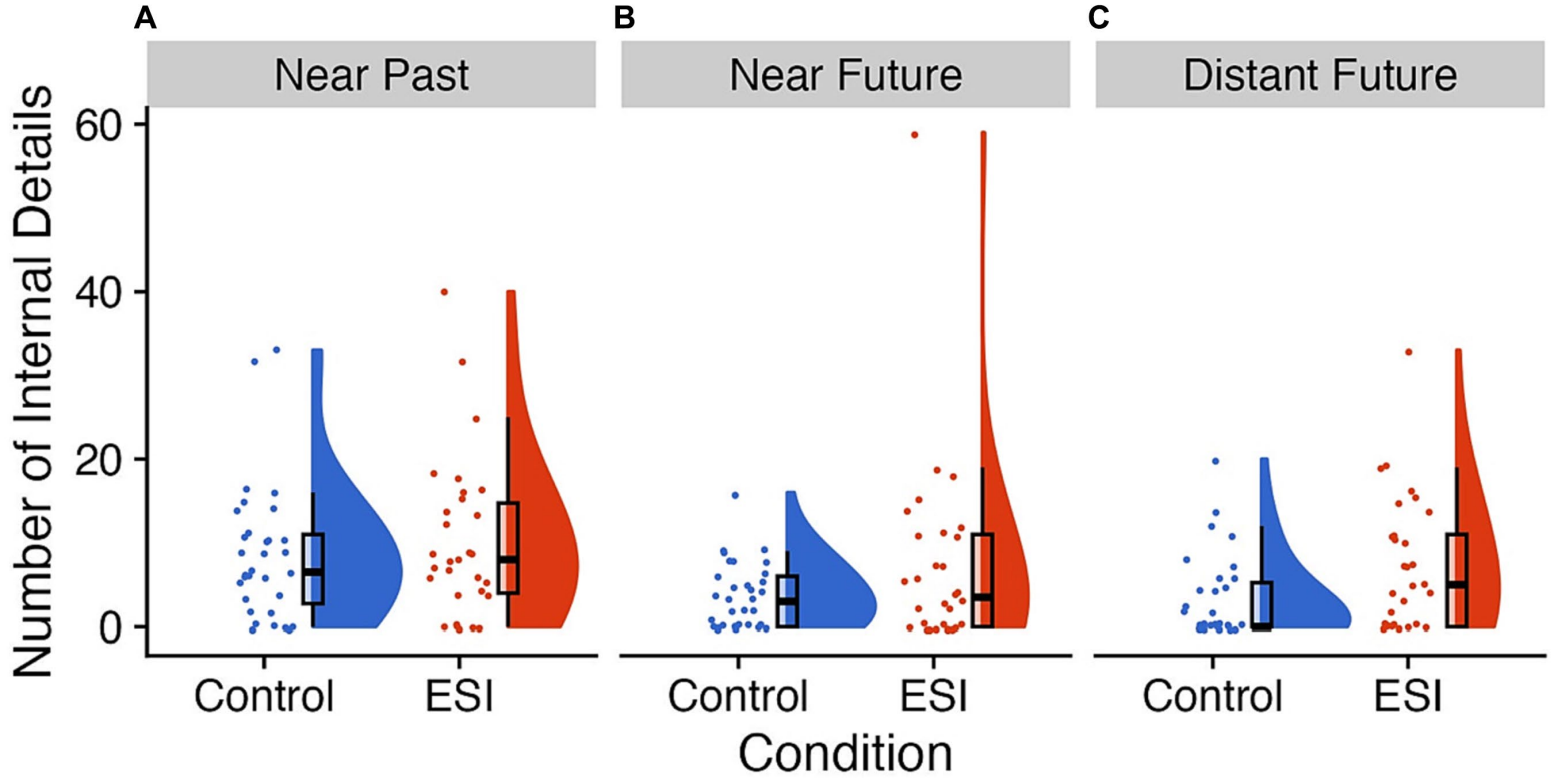
Performance on verbal recollection and imagination of events. Raincloud plots of the number of internal details per trial **(A)** near past, **(B)** near future, and **(C)** distant future with the Control condition on the left of each panel in blue and the ESI condition on the right of each panel in orange. The “clouds” show the probability density function, overlayed with boxplots in black, whereas the “rain” (i.e., dots) represent individual data points. The individual data points were jittered for improved visualization. Prepared in R using RainCloudPlots (Allen et al., 2021).

### 3.2. Outcome measures

#### 3.2.1. Recall/imagination task

##### 3.2.1.1. Episodicity score

We tested the effect of Condition (between-subject: ESI, Control) and Time perspective (within-subject: near past, near future, distant future) on the episodicity score (and also the number of details, see below 3.2.1.2) using a mixed ANOVA. The effect of Condition (ESI: *M* = 3.09, *SE* = 0.16; Control: *M* = 2.79, *SE* = 0.15), *F*(1, 60) =1.14, *p* = 0.290, *η*^2^*_G_* = 0.01, and the Condition’s interaction with Time perspective, *F*(2, 120) = 0.25, *p* = 0.780, *η*^2^*_G_* < 0.01, were not significant. The specificity of the event depended on the temporal orientation, *F*(2, 120) = 11.83, *p* < 0.001, *η*^2^*_G_* = 0.08. Children’s event descriptions were more specific and detailed in the near past (*M* = 3.52, *SE* = 0.17) than near future (*M* = 2.73, *SE* = 0.19), *p* adj. < 0.001, Hedges’ *g* = −0.50, CI 95% [−0.80, −0.25], and near past than distant future (*M* = 2.56, *SE* = 0.19), *p* adj. < 0.001, Hedges’ *g* = −0.56, CI 95% [−0.87, −0.29]. The near and distant future, however, had similar episodicity scores, *p* adj. = 0.453, Hedges’ *g* = −0.10, CI 95% [−0.32, 0.16].

##### 3.2.1.2. Number of internal details

The number of internal details may have a greater range and greater sensitivity to detect an effect of ESI rather than the category of event (e.g., general or specific). A mixed ANOVA showed that children produced a larger number of internal details in total for recalled and imagined events following the ESI (*M* = 7.48, *SE* = 0.79, *n* = 30) than the Control condition (*M* = 4.67, *SE* = 0.50, *N* = 32), *F*(1, 60) = 6.81, *p* = 0.011, *η*^2^*_G_* = 0.05 (see Figure 2). We found a main effect of Time perspective, *F*(2, 120) = 11.26, *p* < 0.001, *η*^2^*_G_* = 0.09. Children generated fewer details for events in the near future (*M* = 4.60, *SE* = 0.66) than the near past (*M* = 8.65, *SE* = 0.93), *t*(61) = 4.06, *p* adj. <0.001, Hedges’ *g* = 0.51, CI 95% [0.31, 0.73], and for events in the distant future (*M* = 4.84, *SE* = 0.75) than the near past, *t*(61) = 3.68, *p* adj. = 0.001, Hedges’ *g* = 0.46, CI 95% [0.25, 0.69]. The near and distant future trials did not differ, *t*(61) = −0.30, *p* = 0.763, Hedges’ *g* = −0.04, CI 95% [−0.29, 0.22]. The interaction between Condition and Time perspective was not significant, *F*(2, 120) = 0.35, *p* = 0.702, *η*^2^*_G_* < 0.01. Nevertheless, we conducted additional planned comparisons to examine the hypothesis that the ESI would have a particularly strong effect on the “near future” trial. In agreement with the main effect of Condition, means were in the direction of children narrating a more detailed specific event following the ESI than Control condition for each of the trials, but the difference was only significant for the distant future trial (see Table 2).

**Table 2:** Pairwise comparisons of internal details between conditions on each trial.

Time perspective	Control		ESI		*p* adj.	Hedges’ *g*
	*M*	*SE*	*M*	*SE*		
Near past	7.47	0.97	9.90	1.60	0.193	−0.33, CI 95% [−0.80, 0.21]
Near future	3.50	0.59	5.77	1.17	0.084	−0.44, CI 95% [−0.92, 0.06]
Distant future	3.03	0.79	6.77	1.22	0.012	−0.65, CI 95% [−1.23, −0.15]

The means and SE are shown after handling outliers as pre-registered (bringing them closer to the mean at *z* ± 2.58) and as in the analyses. Raw values shown in Figure 2.

Data could not be transformed to meet the assumptions of normality and homogeneity of variance. Hence, we also conducted analyses using robust ANOVA to bolster confidence in the results (using the raw data with outliers as is). All significant results above were detected here too (i.e., main effect of Time perspective, differences between past and future trials, and group comparison on distant future), but the main effect of Condition was only approaching significance, *F*(1, 33.01) = 2.78, *p* = 0.105 (all other comparisons, *p* > 0.05 as reported above; see Supplementary Table 4 for full details).

Although we had also pre-registered a coherence analysis based on Seixas Lima et al. (2020), children’s event descriptions were relatively undetailed and thus did not lend themselves well to this more refined analysis.

#### 3.2.2. Prospective memory

Children in the ESI (*log10 transformed as in analysis: M* = 1.16, *SE* = 0.02*; untransformed: M* = 15.20 min, *SE* = 0.81 min, *N* = 32) and Control (transformed as in analysis: *M* = 1.18, *SE* = 0.02; untransformed: 15.51 min, *SE* = 0.67 min, *N* = 34) conditions did not differ in the delay between receiving the Prospective memory instructions and the reminder (i.e., at the end of the session), *t*(64) = 0.67, *p* = 0.505, Hedges’ *g* = 0.16, CI 95% [−0.35, 0.64].

**Figure 3:**
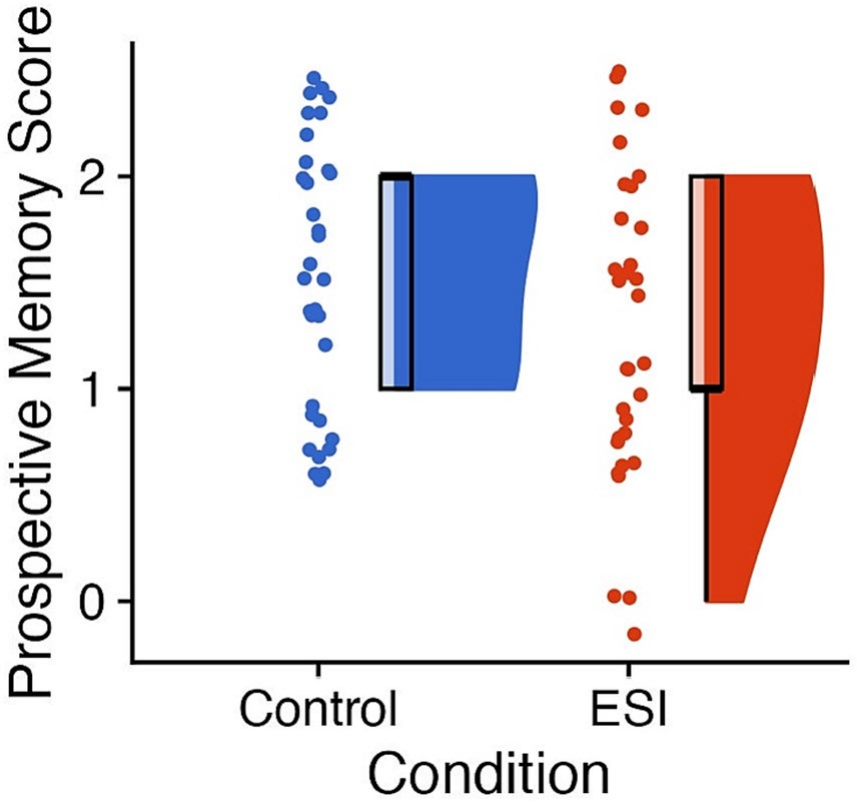
Performance on prospective memory. Raincloud plots of scores on Prospective memory (i.e., remembering to show the favorite thing) with the Control condition on the left of each panel in blue and the ESI condition on the right of each panel in orange. The “clouds” show the probability density function, overlayed with boxplots in black, whereas the “rain” (i.e., dots) represent individual data points. The individual data points were jittered for improved visualization. On the y axis, 0 = prospective memory failure, 1 = remembering the action after prompt, and 2 = remembering the action immediately. Prepared in R using RainCloudPlots (Allen et al., 2021).

A chi-square test showed no significant difference in performance on the Prospective memory task between the two conditions, χ ^2^(2, *N* = 65) = 3.29, *p* = 0.193, Cramer’s *V* = 0.29 (see Figure 3).

#### 3.2.3. Delay of gratification

**Figure 4:**
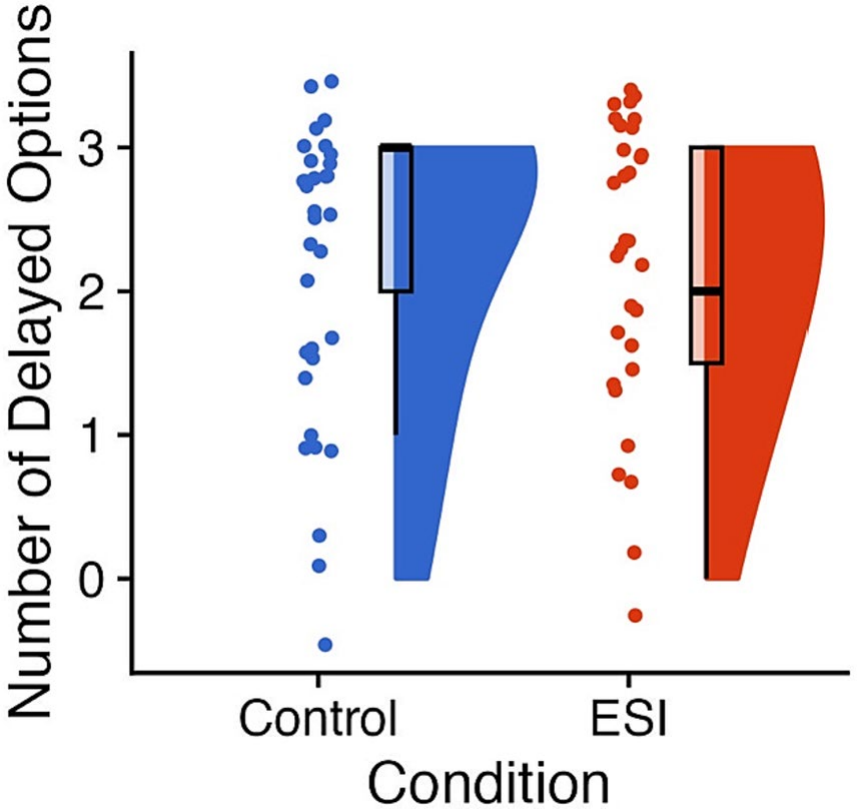
Performance on Delay of gratification. Raincloud plots of scores on Delay of gratification (i.e., total number of delayed options; maximum of 3) with the Control condition on the left of each panel in blue and the ESI condition on the right of each panel in orange. The “clouds” show the probability density function, overlayed with boxplots in black, whereas the “rain” (i.e., dots) represent individual data points. The individual data points were jittered for improved visualization. Prepared in R using RainCloudPlots (Allen et al., 2021).

Children in the ESI condition (dog = 53.13%, cat = 46.88%) and Control condition (dog = 47.06%, cat = 52.94%) preferred dog and cat versions of the task similarly, χ ^2^(1, *N* = 66) = 0.06, *p* = 0.806, *φ* = 0.03. The ESI (*Mdn* = 2.00, *M* = 2.13, *SE* = 0.17, *N* = 31) and Control (*Mdn* = 3.00, *M* = 2.21, *SE* = 0.18, *N* = 33) conditions did not differ on the Delay of gratification task, *W* = 547.50, *p* = 0.606, *r* = 0.07 (see Figure 4).

#### 3.2.4. Picture-book task

##### 3.2.4.1. Behavioral measure

**Figure 5:**
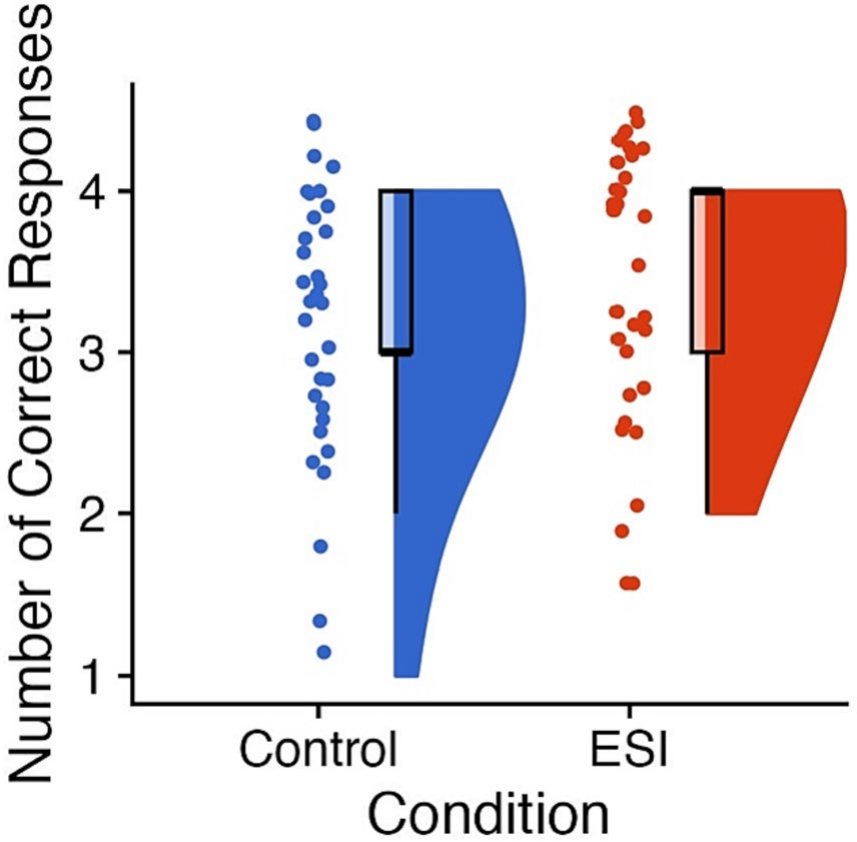
Performance on the behavioral measure of the picture-book task. Raincloud plots of scores on the Picture-book task (i.e., total number of correct responses; maximum of 4) with the Control condition on the left of each panel in blue and the ESI condition on the right of each panel in orange. The “clouds” show the probability density function, overlayed with boxplots in black, whereas the “rain” (i.e., dots) represent individual data points. The individual data points were jittered for improved visualization. Prepared in R using RainCloudPlots (Allen et al., 2021).

A Mann–Whitney test showed no significant difference between children in the ESI condition (*Mdn* = 4.00, *M* = 3.41, *SE* = 0.13, *N* = 32) and the Control condition (*Mdn* = 3.00, *M* = 3.12, *SE* = 0.15, *N* = 33) in terms of the number of correct items selected on the Picture-book task, *W* = 432.50, *p* = 0.175, *r* = 0.17 (see Figure 5).

##### 3.2.4.2. Verbal measure

Children’s verbal explanations for their item choices were scored based on whether they contained a future term and referenced a future physiological state. A Mann–Whitney test showed no significant difference between children’s explanations in the ESI (*Mdn* = 1.88, *M* = 1.84, *SE* = 0.12, *N* = 32) and Control (*Mdn* = 2, *M* = 1.91, *SE* = 0.11, *N* = 33) conditions, *W* = 572.50*, p* = 0.560, *r* = 0.07.

## 4. Discussion

EFT has significant ramifications for decision-making, creativity, and problem-solving; as such, finding ways to enhance EFT in children, even temporarily, warrants investigation. Our adaptation of a future-oriented episodic specificity induction for young children (6 and 7 years old) was successful in facilitating the imagination of a richly detailed future event, as reflected in the number of internal details during the induction and subjective measures of vividness. All children imagined tomorrow’s breakfast, an event for which children typically have control (Quon and Atance, 2010), allowing for greater standardization of the procedure and consistency across children. Like Chernyak et al. (2017), we asked about a daily event that was anchored in a relatively near future (i.e., tomorrow morning), but also focused on drawing out a highly detailed description of a single event. The control condition was well matched to the ESI condition overall. It unfolded within a social interaction with the researcher who asked questions about a picture showing a child having breakfast. Hence, the format and questions were similar between the two conditions, but the control condition did not involve the construction of an event nor a future-oriented component. Children described more detailed specific events on our Recollection/Imagination outcome task following the ESI than the control condition. Follow-up analyses showed the effect was the strongest when imagining an event in the distant future. However, our significant effect (contrasting conditions on trials overall) was not retained when using the robust ANOVA. We also did not find a difference between the ESI and Control conditions in terms of the episodicity scores in the Recollection/Imagination task. In this sense, the effects of our ESI were weaker than anticipated. Indeed, we expected an effect size larger than *η*^2^*_p_* = 0.11 (based on Chernyak et al., 2017), but obtained an effect size of only *η*^2^*_G_* = 0.05. The additional outcome tasks, administered later in the study, also did not show benefits of ESI. Although there might be ways to improve how an ESI is administered to young children, a very real possibility that has recently been supported by empirical work in other areas is that there are limited benefits of relying on EFT to ameliorate performance on future-oriented tasks in young children. For example, immediately preceding “cues” to think about future events did not help (and maybe even harmed) performance on a Delay of gratification task in children aged 4–5 years (Burns et al., 2021) and 8–12 years old (Canning et al., 2023). This contrasts with the positive effects seen in adults (Ballance et al., 2022; Rösch et al., 2022).

It is unclear when an ESI could be expected to produce sufficiently strong effects, if indeed ESI depends on a mature “episodic” system. The hippocampus, a critical structure for episodic constructive processes, continues to develop along with episodic memory into adolescence (Botdorf et al., 2022). Consistent with this, children in our study described specific events that were sparse in details (mean of 3 to 10 details per trial/condition) on our Recollection/Imagination outcome task, often falling short of conveying episodic construction. That is, many of the events children provided read more like a “to-do list” than a true pre-experiencing of a future event. Thus, aiming to achieve a criterion of episodic richness in children during ESI may be too cognitively taxing as illustrated by the duration of the ESI induction (~14 min on average). Language and semantic demands, as well as episodic demands, may also be greater in children than adults as exemplified in this participant’s statement: “It’s pretty hard to explain all this stuff I do that I wanna do.” In this sense, ESI could have depleted the cognitive resources and minimized positive effects of ESI on subsequent tasks, including the Recollection/Imagination task. A less stringent criterion or a focus on the most vividly imagined aspects (i.e., scene, actions, self) may help to avoid fatigue. The rating of visual clarity along with inspection of the data suggest that imagining other people in the future was particularly challenging during our ESI. In adults, the ESI has been adapted to shift to emphasize either spatial or temporal information, altering the downstream effects of ESI (Sheldon et al., 2019). Adapting ESI for children may also entail tailoring the set of themes and questions to the mechanisms thought to underlie the outcome task of interest. For example, episodic elaborations on actions might be particularly suitable to foster prospective memory improvements (Leech et al., 2019; Cottini et al., 2021), whereas the elaboration of thoughts and feelings might benefit emotion regulation.

Chernyak et al. (2017) and Leech et al.’s (2019) success in improving EFT may come in part from capitalizing on the burgeoning general and personal semantic memory systems (e.g., knowledge about the world, personal identity, and personal events) rather than the episodic memory system. Indeed, episodic memory depends on semantic memory (Greenberg and Verfaellie, 2010), and knowledge “scaffolds” episodic future thinking (D’Argembeau and Mathy, 2011; Irish and Piguet, 2013). The developmental literature has long recognized the role of the development of the self and semantic memory in facilitating the development of episodic memory (Fivush, 2011), and to some extent EFT (Martin-Ordas et al., 2014). Thus, future-oriented interventions might engender benefits because of their emphasis on strengthening relevant knowledge structures (e.g., reactivating information, adding new information, and/or creating new links between pieces of information). Accordingly, relying on children’s further developed semantic memory might help to constrain intervention duration and sustain engagement. Hence, younger children may benefit most from a semantic specificity (or semantic richness) induction to bolster knowledge relevant to the future. A “semantic specificity induction” would have antecedents in gist-based inductions (Grilli et al., 2019) and activities to develop a stronger sense of self (Jacquez et al., 2020; Van der Aar et al., 2022). Research on construal level theory further indicates that a more abstract versus concrete mode of thinking can foster cognitive benefits (Trope and Liberman, 2010; Liberman and Trope, 2014), including for future thinking in young children (Bélanger et al., 2014; Lee and Atance, 2016). Alternatively (or conjointly), projecting oneself into the future, even briefly and with relatively little contextual specificity might suffice to enhance performance on some future-oriented tasks in children (e.g., Cottini et al., 2021).

The cognitive load of our ESI could also perhaps be reduced through a shift to past-oriented ESI, given that future thinking in general is more demanding and less rich than episodic memory (Coughlin et al., 2014; Wang et al., 2014). Similarly, we found that children generated more details and more specific events on past-oriented than future-oriented trials during the Recollection/Imagination outcome task. A past-oriented ESI, like the original version (Madore et al., 2014), might carry a unique advantage to encourage episodic elaboration at lower cognitive cost in children. Moreover, the use of a video for the encoding of a past event would simplify the standardization of the procedure. Importantly, even though a future-oriented ESI appears to hold modest promise in comparison with other future-oriented interventions (Chernyak et al., 2017; Leech et al., 2019), an ESI could still produce some benefits in some children and at some ages. Our sample size did not allow for the examination of individual factors, but the high variability indicates ESI could be particularly advantageous for some children. Thus, investigation is still warranted to delineate the relative importance of episodic elaboration, semantic elaboration, projecting into the past/future, and individual characteristics.

As pointed out in the introduction, cognitive development creates particular challenges for future-thinking interventions. There are also several degrees of freedom when designing an ESI suitable for young children. For example, drawing could be leveraged as a tool to facilitate episodic elaboration and stimulate engagement (Chernyak et al., 2017; Tran et al., 2022). On a cautionary note, however, initial in-person piloting (pre-pandemic) revealed that a drawing and magnet activity can encourage descriptions of unrelated and unrealistic events rather than facilitate the elaboration of a single future event. The event selected for use in the ESI offers yet another degree of freedom. In this study, the pandemic context (e.g., during lockdown) and online administration severely constrained the type of events that could be imagined in the future. Subsequent research should examine a broader range of events (both during the induction and outcome tasks). The person administering ESI constitutes another moving piece. Although researchers have administered ESI in adults, parents could be advantageous allies to help elicit specific events in children (Wang et al., 2019; Shin et al., 2020). The common ground (*cf.* Brown-Schmidt and Duff, 2016) could facilitate the communication and exploration of episodic elements in future events. Therefore, another avenue for a child-friendly ESI may be to involve parents, akin to Wang et al. (2019), but using a short-duration intervention. Lastly, initial in-person piloting guided the choice of an interview-style ESI with children. On the one hand, ESI influences the “construction” rather than “elaborative” phase when imaging future events (Madore et al., 2016b), suggesting less support would be desirable. On the other hand, the developing communicative and episodic abilities could justify a heightened level of scaffolding. Future research will need to establish the optimal level and format of support.

Future thinking permeates the daily lives of children and their parents: A parent prepares a glass of milk at the request of their child only to discover minutes later that their child has lost interest in the drink. A child does not put a pet back in its cage, failing to foresee the damage that could ensue. A child does not anticipate the boredom of a rainy day on a camping trip and fails to bring indoor games. Educational pursuits could inspire countless additional examples. The improvement of future thinking, even on a temporary basis, holds promise for immediate and long-term benefits. Our child-friendly ESI demonstrated potential immediate benefits on the recollection and imagination of events through an increase of details. In contrast, the minimally-verbal outcome tasks offered no hints of improvement, possibly due to an order effect or the cognitive functions involved in the tasks. Therefore, we found no indication that this version of ESI could translate to better choices for the future or remembering actions in children. Even though we conclude on the limited benefits of relying on the episodic memory system at young ages, our findings along with others (e.g., Chernyak et al., 2017; Leech et al., 2019; Wang et al., 2019) support the exciting prospect of achieving EFT improvement in the daily lives of children.

## Data availability statement

The datasets presented in this article are not readily available because of ethical restrictions. End-stage data will be made available. Requests to access the datasets should be directed to AT, atang027@uottawa.ca.

## Ethics statement

The studies involving humans were approved by Health Sciences and Sciences Research Ethics Board of the University of Ottawa. The studies were conducted in accordance with the local legislation and institutional requirements. The Ethics Committee/Institutional Review Board waived the requirement of written informed consent for participation from the participants or the participants’ legal guardians/next of kin because of the online administration; verbal consent/assent were obtained.

## Author contributions

AT, OG, JA, GA, and CA: conceptualization and methodology. AT: formal analysis and visualization. OG and JA: investigation. CA: resources, supervision, and funding acquisition. AT, OG, JA, and GA: data curation. AT and OG: writing – original draft. GA, OG, JA, and CA: writing – review and edition. AT and CA: project administration. All authors contributed to the article and approved the submitted version.

## Footnotes

^1^ https://osf.io/5t2bk/
