# Supplementary material for: Using an episodic specificity induction to improve children’s future thinking
Source: Front Psychol. 2023 Oct 19;14:1249090. doi: 10.3389/fpsyg.2023.1249090 (PMC10622774; doi:10.3389/fpsyg.2023.1249090)
Supplement: Supplementary file 1 [file Data_Sheet_1.docx]

Supplementary Material

Using an Episodic Specificity Induction to Improve Children’s Future Thinking

Annick F. N. Tanguay^1†^, Olivia Gardam^1†^, Jane Archibald^1^, Gladys Ayson^1^, Cristina M. Atance^1*^

*** Correspondence:** Corresponding Author: [atance@uottawa.ca](mailto:atance@uottawa.ca)

# Supplementary Tables

## Table 1

*Questions in Each Condition*

| ESI | Control |
| --- | --- |
| Surroundings | |
| 1. Tell me where you will be?    1. Can you tell me more about where this is?    2. Where are you going to eat breakfast?   *A specific location must be named (kitchen, dining room, bedroom, restaurant). It should also be clear where breakfast is going to be eaten.*   1. Tell me more about this (location), what does it look like?    1. Can you tell me more about what the (location) looks like?    2. Tell me more about (detail of location, object, etc.). Is it big or small? What is its colour? Where is it? Is there anything else on your walls? Is there anything else around you?    3. What will you be eating for breakfast? Tell me more about this. What does it look like?   *A clear description is when you feel that you can visualize the space as if you were in the location yourself. You might not see all minute details, but you have the gist. All locations that were named in 1 (if more than one) should be described. Further, you should also have a clear picture of the breakfast itself (e.g., what is it, is it good?).*   1. What is the weather like outside? Is it any different from today?    1. Can you tell me more about the weather? How is it outside?   *A clear description is when you know the weather outside (e.g., is it sunny? Cloudy? Dark? Cold? Hot? Rainy?). The child should also speak to any difference from today. Note that it’s OK if there are no differences.*   1. Will anyone else be there? Will there be any pets there?    1. Will there be anyone else? Or any other pets?    2. *In the case that no one will be there, confirm*: Tomorrow morning, you will be having breakfast all on your own?   *A clear description is when the child has clearly specified whether there is no one or some people/pets. If there are people/pets, they should be identified somehow. It’s not necessary to have names, but you should get a sense of how many unique people/pets there are.* | What is the boy/girl doing?  Where is the boy/girl?  Can you tell me more about this place? What does it look like?  Where is the boy/girl eating breakfast? What does it look like?  Tell me more about (object).  Is there anything on the walls?  What is the boy/girl eating for breakfast?  Tell me more about the breakfast. What does the breakfast look like? Are they drinking something? What does that look like?  What is the weather like outside?  Is there anything else around the boy/girl?  Is there anybody else or any animal in the picture? |
| Appearance | |
| 1. What will you be wearing?    1. Tell me more about your outfit. Is it a dress/skirt/pants/sweater/t-shirt? How does it look? What will be the colour of your outfit?   *A clear description is when the child seems to describe a specific outfit.*   1. What will your hair look like?    1. Tell me more about what you hair will look like.   *A clear description is when you get a sense of style or general look. No need to prompt for differences.*   1. Will you look any different from today?    1. Are there any other differences in how you will look?   *A clear description is when the child mentions at least one difference. We should understand what the difference is, but no need to prompt for additional details otherwise.*   1. How will you be feeling?   *A clear description is when the child identified at least one feeling. Feelings are loosely defined (e.g., tired, happy, hungry, excited).*   1. You mentioned (people/pets) would be there. How will they look?    1. Tell me more about how they will look: What will they be wearing?    2. What will their hair look like?    3. How will they be feeling? (*choose 1 of these 3 to ask).*   *A clear description is when the child seems to be representing people tomorrow, as a specific look or feeling would suggest. However, do not aim to obtain the same level of detail as you did in 5-8 for the child himself/herself. One or two details per person is enough.* | What is the boy/girl wearing?  What does his/her hair look like?  Is the boy/girl happy or sad?  What does the animal look like? |
| Actions | |
| 1. It is breakfast time. Tell me everything that will be happening.    1. Tell me more!    2. Tell me more about your breakfast. What does it smell like? What does it taste like?   *A clear description is when the child has described the sequence of 2-3 actions (e.g., my dad prepared pancakes, I will eat them and it will be delicious, my brother will tease me). This “Action” section gives the opportunity to put together all the previously mentioned details into a coherent story. 10 & 11 focus on what happens before/during breakfast (if applicable) and 12 on what happens after.*   1. Will there be anything else happening around you?    1. You mentioned (object, person, pet), what are they doing?    2. Are you doing anything with them?   *A clear description is when the child has given you information that shows you how the objects/people/pets (etc.) fit within the narrative. It is OK if the objects/people/pets (etc.) are passive (i.e., not interacting with the child), but you could ask about interaction to get more details.*   1. Now you’ve finished eating, what will you do next?    1. Tell me about anything that is going to happen after breakfast tomorrow. What are you going to do next?   *A clear description is when the child has given a sequence of 1-3 actions. The actions must not be breakfast related and do not need to be close in time. Here we want to push a bit more the “episodic” quality of the event and get a sense of the breakfast unfolding in the context of a unique day. Nevertheless this section is coarser grained than the breakfast mini-event itself (which we expect to be highly detailed).* | What is happening in the picture?  Is there anything else happening around the boy/girl?  What is the animal doing? |

*Note*. For ESI, the researcher always asked the initial question (numbered). The researcher proceeded with one or many follow-up questions (with letters) in cases of unclear or rudimentary responses. Guidelines (in italics) set the bar for an acceptable level of details.

## Table 2

*Description and Examples of Episodicity Scores*

| Score | Description | Example | Actual Examples from Participants |
| --- | --- | --- | --- |
| 0 | No event or a general statement | I like ice cream | “Next year I will be in third grade.” (distant future) |
| 1 | The event is vague, or recurrent, with little to no spatial or temporal details | I will eat ice cream | “We’ll have family, we’ll have family movie.” (near future) |
| 2 | Generic event with spatial and/or temporal details | Eating ice cream at a restaurant for dinner | “(…)Um, I, I, think I’m gonna um, go there again, and, and get the COVID-19 shots sometime.” (near future) |
| 3 | Specific event with precise spatial and temporal details | I will eat ice cream for dessert at my favourite restaurant next week | “(…)after tomorrow I’m gonna like present like, um, like s– Like, um, a homework online about China.” (near future) |
| 4 | As in 3, with additional contextual details (e.g., thoughts, emotions, or imagery) | I will go to a restaurant with my family for dinner next week. I will be really happy because I’ll get to have chocolate ice cream with pink and purple sprinkles, my favourite dessert. It will be delicious. | “Well, we could go to a family restaurant named, um, [Name of Restaurant], and also the building is going to be all red. (…) Um, people are going to be wearing uniforms that are like overalls and they have a picture of a dog on them.” (near future) |
| 5 | As in 4, with a logical progression | Next week, in the evening, I will put on a nice outfit, then we will pick up my aunt and uncle at their house, and then I will go to the restaurant with my family and order chocolate ice cream. We will drive home and watch a movie before bedtime. | (…) “And then, and then after, and then after when I went to school, um, and it was the end of the day, um, my mom and dad wasn’t there, and then I said, “I knew it! I’m going in the school bus”. And then after, and then after, um, and then after, I went in the school bus and I was like, “Sheesh, it’s crowded in here”, and then after I saw my friend, I’m like, “Bye, see ya later” (near future) |

## Table 3

*Picture-Book Task Nature Scenes and Item Choices*

| Nature Scene | Physiological state | Correct item | Semantically viable item | Semantically incorrect item #1 | Semantically incorrect item #2 |
| --- | --- | --- | --- | --- | --- |
| Rocky stream | Wet feet | Rain boots | Flip flops | Paddle | Goggles |
| Snow-covered path | Cold | Tuque | Baseball cap | Snow globe | Ice cube |
| Mountain | Hunger | Sandwich | Bowl of soup | Sticks | Plant |
| Waterfall | Wet | Raincoat | Bath robe | Soap | Rubber duck |

*Note*. Pictures of the scenes and items were displayed along with the verbal instructions.

## Table 4

*Results of Robust Tests for the Number of Internal Details on the Recall/Imagination task*

| *Effect or Contrast* | *Statistics* |
| --- | --- |
| *ANOVA (bwtrim in WRS2; Mair et al., 2022)* | |
| *Main effect of Condition* | *F*(1, 33.01) = 2.78, *p* = .105 |
| *Main effect of Time* | *F*(2, 28.03) = 9.10, *p* = .001 |
| *Interaction between Condition and Time* | *F*(2, 28.03) = 0.94, *p* = .402 |
| *T-test (yuen in WRS2;Mair et al., 2022)* | |
| *ESI vs. Control – Near Past trial* | *t*(34.52) *=* 0.69*, p =* .497*,* effect size *=* 0.13, trimmed mean = -1.37, CI 95% [-5.41, -2.67] |
| *ESI vs. Control – Near Future trial* | *t*(28.99) *=* 0.72*, p =* .478*,* effect size *=* 0.15, trimmed mean =  *-*1.22, CI 95% [-4.70, 2.25] |
| *ESI vs. Control – Distant Future trial* | *t*(23.26) *=* 2.11*, p =* .046*,* effect size *=* 0.41, trimmed mean =.  *-*4.01, CI 95% [-7.93, -0.08] |
| *T-test (yuen in WRS2;(Mair et al., 2022)* | |
| *Near past vs. Near future* | *t*(67.93) *=* 3.58, *p* < .001*,* effect size *=* 0.45, trimmed mean = -4.53, CI 95% [-7.05, -2.00] |
| *Near past vs. Distant future* | *t*(73.48) *=* 3.40*, p =* .001*,* effect size *=* 0.40, trimmed mean = -4.71, CI 95% [-7.47, -1.95] |
| *Near future vs. Distant future* | *t*(70.57) *=* 0.15*, p =* .878*,* effect size *=* 0.02, trimmed mean = -0.18, CI 95% [-2.58, 2.21] |

# References

Mair, P., Wilcox, R., and Patil, I. (2022). Package ‘WRS2.’ Available at: https://cran.r-project.org/web/packages/WRS2/.
